# A New Remedy for Asthma

**Published:** 1887-11

**Authors:** L. J. Graham

**Affiliations:** Henderson, Texas


### CORRESPONDENCE.

### A NEW REMEDY FOR ASTHMA—ANTIPYRINE.

HENDERSON, TEXAS, October 12, 1887.

*F. E. Daniel, M. D., Editor, etc., Austin, Texas.*

DEAR SIR: Allow me to call the attention of the profession to this medicine as a remedy in this most distressing of diseases. I have not observed, in my reading, any mention of antipyrine as a remedy for asthma; and I will here mention a case that I treated with this remedy with marked success.

A short time since, I was called to see Mr. I., living six miles in the country. He is 50 years of age. The messenger did not intimate to me what was the nature of the trouble; but the call was urgent. I went at once, and found the poor man suffering intensely from an attack of asthma. I had never treated him before, and hence did not know what kind of a case I would find, and therefore did not have with me any of the usual remedies used in the treatment of asthma, but was armed *cap a pie* with medicines used in the treatment of various fevers we meet with in this section of the country. Among these I had antipyrine. I had been reading of the good effects of this remedy in the various neurotic troubles, migraine, etc., and the idea struck me that asthma, being a nervous affection, the remedy would prove of benefit in this case; so I gave him fifteen grains of the drug, with directions to repeat the dose in an hour. I remained with him till some time after the second dose was given. After this, the improvement was very perceptible. I then left, with directions to repeat the dose as occasion required. He came to town in a few days, and reported that the medicine relieved him in a short while. Since then, antipyrine has been a regular "stand-by" with him. He visited town to-day to lay in another and larger supply, telling me that antipyrine was "a good medicine for asthma."

This one case, of course, will not establish antipyrine as one of the standard remedies in the treatment of asthma; and this letter is not written for that purpose, but it is to direct attention to it, with the hope that others of the brethren will give it a fair trial.

With much respect,

I am yours,

L. J. Graham, M. D.

